# African swine fever virus NAM P1/95 is a mixture of genotype I and genotype VIII viruses

**DOI:** 10.1128/mra.00067-24

**Published:** 2024-03-25

**Authors:** Lynnette C. Goatley, Graham L. Freimanis, Chandana Tennakoon, Armanda Bastos, Livio Heath, Christopher L. Netherton

**Affiliations:** 1 The Pirbright Institute, Woking, United Kingdom; 2 Department of Zoology & Entomology, Faculty of Natural and Agricultural Sciences, University of Pretoria, Pretoria, South Africa; 3 Department of Veterinary Tropical Diseases, Faculty of Veterinary Science, University of Pretoria, Pretoria, South Africa; 4 Agricultural Research Council-Onderstepoort Veterinary Institute, Onderstepoort, South Africa; Katholieke Universiteit Leuven, Leuven, Belgium

**Keywords:** African swine fever virus, mixed population, genome, Illumina MiSeq, oxford nanopore

## Abstract

African swine fever virus causes a lethal hemorrhagic disease of domestic pigs. The NAM P1/1995 isolate was originally described as *B646L* genotype XVIII; however, full genome sequencing revealed that this assignment was incorrect.

## ANNOUNCEMENT

**Fig 1:**
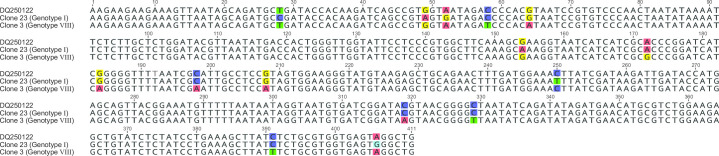
Alignment of the 3´ end of the ASFV *B646L* gene from two clones obtained from a NAM P1/95 sample and sequence DQ250122. Differences between DQ250122 and the two clones are highlighted.

African swine fever virus (ASFV) NAM P1/1995, family *Asfarviridae*, genus *Asfivirus*, was originally classified as *B646L* genotype XVIII (1) and is the only known virus of this genotype. The isolate, originally obtained from a domestic pig in Namibia was sequenced to improve our understanding of ASFV genetics. Virus was propagated on macrophage cultures (2) until 90%–100% cytopathic effect was observed and cell debris was removed by centrifugation (1,000 × *g*, 5 min, 4°C). Virus particles were then concentrated by ultracentrifugation (13,600 × *g*, 90 min, 4°C), treated with TURBO DNase and then DNase inactivation reagent (ThermoFisher). Genomic DNA was prepared using a MagAttract HMW extraction kit (Qiagen), isothermally amplified using REPLI-g (Qiagen), quality assessed using TapeStation (Agilent) to confirm the presence of high molecular weight fragments (20 to 60 kbp), and quantified using a Qubit dsDNA BR assay kit (ThermoFisher). Five hundred nanograms of DNA was prepared using the DNA Prep Kit (Illumina) for an Illumina MiSeq 600 cycle v3 cartridge or 2 µg DNA was individually barcoded (NBD104 and LSK109) for Minion (MIN-101b) sequencing on a 9.4.1 MinION flow cell, following the manufacturer’s instructions. Illumina adaptors and reads with a quality score <30 were removed with Trim Galore (0.6.10), and Nanopore adaptors and reads with a quality score <10 were removed with Chopper (0.5.0) and Porechop (0.2.4) using default parameters. Contigs were assembled from both Illumina and Nanopore reads using SPAdes (3.15.3) with --isolate option, and reads were then mapped to the final assembly in Geneious Prime (2023.2.1) to identify single nucleotide polymorphisms and correct assembly errors. Surprisingly, pairwise Geneious Prime (2023.2.1) alignment revealed that the *B646L* gene, which encodes for the major capsid protein p72, was identical to the genotype VIII reference strain Malawi Lil 20/1 (AY261361). Therefore, we Sanger sequenced (3) the original biobanked sample from the Pirbright reference collection of NAM P1/1995 and identified ambiguities consistent with a mixed population of genotype I and genotype VIII viruses. Virus cultures containing genotype I (clone 23) and genotype VIII (clone 3) viruses were generated by limit dilution (2) and the *B646L* sequences from the two clones compared to the original NAM P1/1995 *B646L* sequence DQ250122 (Fig. 1). This confirmed that the assignment of genotype XVIII to NAM P1/1995 was incorrect and that the original sample was a mixed population of viruses.

NAM P1/1995 clone 3 was then subjected to full genome sequencing as described above, generating 4,705,288 Illumina and 16,388 Nanopore reads that resulted in an assembly of 185,514 bp that was 99.064% identical to the sequence of Malawi Lil 20/1 (Table 1). The final assembly was initially annotated using genome annotated transfer utility (4) with Malawi Lil 20/1 as a reference. Multigene family open reading frames (ORFs) were assigned as per Imbrey et al. (5) and other ORFs assignations by reference to ASFV transcription maps (6, 7). Our suggestion for those working on ASFV discovery is that genotype XVIII is retired, and as and when new genotypes are identified, they are numbered from XXV onward.

**TABLE 1:** Details of NAM P1/95 sequences

Sequence	Length (bp)	Composition (%GC)	Illumina coverage (min–max)	Nanopore coverage (min–max)	Accession number
Clone 3 partial *B646L* sequence	411	41.4	NA^*a*^	NA	PP107959
Clone 23 partial *B646L* sequence	411	43.1	NA	NA	PP107958
Clone 3 genome assembly	185,514	37.9	150 to 20,362	4 to 64	PP107957

^*a*^ NA, not applicable.

## Data Availability

Accession numbers for the genome assembly of NAM P1/1995 Clone 3 and the partial B646L sequences of NAM P1/1995 Clone 3 and Clone 23 are PP107957, PP107959 and PP107958 respectively and the raw data are available in BioProject PRJNA1063215 and SRX23149111, SRX23149112, SRX23338677 and SRX23338678.
